# Quality Assessment of Greek-Style Set Yoghurt Fortified with Extracted and Dried Australian Native Fruit, Illawarra Plum

**DOI:** 10.3390/foods13142185

**Published:** 2024-07-11

**Authors:** Rebecca McCullum, Md Saifullah, Michael Bowyer, Quan Vuong

**Affiliations:** 1 College of Engineering, Science and the Environment, University of Newcastle, 10 Chittaway Road, Ourimbah, NSW 2258, Australia; michael.bowyer@newcastle.edu.au (M.B.); vanquan.vuong@newcastle.edu.au (Q.V.); 2 Centre for Food Innovation, Tasmanian Institute of Agriculture, University of Tasmania, Newnham, TAS 7248, Australia; md.saifullah@utas.edu.au

**Keywords:** yoghurt, additive, natural, fruit, quality

## Abstract

The Illawarra plum (IP) is native to Australia and has been used as a bush food for centuries. With rich phytochemicals and natural pigments, IP has the potential to be an added ingredient to improve the physicochemical properties of food, including yoghurt. This study prepared dried IP powders through vacuum drying (VD) and freeze drying (FD), produced extracts from these dried powders, and fortified them into Greek-style set yoghurt. The changes in colour, pH, titratable acidity (TA), syneresis, total soluble solids (TS), and phytochemicals were measured throughout a chilled storage period of 28 days. The results showed that FD and FD extract could provide a stable, distinct pink colour to yoghurt. IP powders and their extracts improved TS content and, thus, the consistency of yoghurt. Compared to the control, VD, FD, and FD extract of IP did not significantly affect the level of acidity or syneresis after 28 days of chilled storage. Yoghurt fortified with FD and FD extract had the greatest level of phenolics, anthocyanins, and radical scavenging antioxidant activities. This study revealed that IP powders and their extracts can positively improve the physicochemical properties of Greek-style set yoghurt. FD powder is recommended over its extract to fortify this yoghurt, as it can be cost-effectively prepared by freeze drying and then grinding the fresh fruit into powder. Future studies are needed to evaluate other variables in yoghurt preparation, including the concentration of IP and strains of yoghurt culture. Further, the consumer perception of sensory quality and acceptability of yoghurt fortified with FD IP powder should be investigated.

## 1. Introduction

Yoghurt is a dairy product made by the lactic acid fermentation of milk, and its product range is constantly expanded through enrichment with various additives [1]. Common additives include stabilisers, acidity regulators, gelling agents, flavourants, thickeners, colours, and preservatives [2]. Additives can increase the sensory and physicochemical properties of yoghurts, such as acidity, total solids, texture, colour, and nutraceutical composition, which are important quality aspects of yoghurts. Acidity contributes to the flavour, stability, and safety of the yoghurt during its shelf life. The total solids measurement can indicate the composition of proteins, sugars, minerals, and similar non-fat components. The texture of the yoghurt can be altered by emulsifiers, which enhance creaminess and prevent fat separation. Additives such as stabilisers and thickeners can prevent the separation of whey (syneresis) and maintain the desired mouthfeel [2]. Syneresis is considered an important quality indicator of yoghurt [3]. Syneresis may negatively affect consumer perception of yoghurt by impacting body and texture characteristics [4]. Colourants can be added to yoghurts to intensify or enhance the visual appeal of yoghurt. Nutraceutical composition can affect the growth of probiotics such as lactic acid bacteria and deliver health benefits [5]. Probiotics are often sensitive to oxygen, and the addition of antioxidants can lower the redox level to enhance the growth of beneficial bacteria [6].

There is an increasing demand for natural additives and antioxidants that replace synthetic colourants, flavours, stabilisers, and preservatives [7,8]. While milk and dairy products have their own antioxidant content, the amount of phenolic compounds in dairy products is restricted, and yoghurt is not considered a major source of phenolics. This could be due to decomposition during processing or the effect of sanitising agents on equipment during production [9]. Thus, novel sources of phenolics are being used to fortify yoghurts and improve their functional profile. However, negative effects on the yoghurt’s stability have been reported from fortification with certain fruits, with each fruit affecting yoghurts differently, such as increased syneresis from the addition of blueberry, aronia or grapes, or increased acidity from added kiwi, cherry, or peach [10,11].

Australia is a large and climactically diverse island continent with numerous native fruits which have been used as bush food and medicine by Indigenous Australians for tens of thousands of years. The Illawarra plum (IP), *Podocarpus elatus*, is endemic to the east coast of Australia and fruits from March to July (Southern hemisphere) [12,13]. IP fruit is red-purple in colour and is rich in phenolic antioxidants and anthocyanins. Preliminary data revealed that IP fruit exhibits antioxidant and antimicrobial activity in vitro. As well as having protective properties against leukemic, gastric, and colon cancer cell lines, IP offers beneficial chemotherapeutic effects [14,15,16,17,18]. IP fruit is dried under different conditions, and their extracts have differences in composition [19]. As such, this study investigated the impact of dried IP or IP extracts on the physicochemical properties of the yoghurts. The outcomes of the study clarified the potential use of the Illawarra plum as a natural food additive or functional ingredient and justified the level of postharvest processing required to deliver optimal benefit to yoghurt quality.

## 2. Materials and Methods

All chemicals used in this study were analytical grade. Methanol, ethanol, vanillin, and NaOH were purchased from Merck (Darmstadt, Germany). Folin–Ciocalteu’s reagent, Na_2_CO_3_, NaNO_2_, CH3CO2Na, AlCl_3_·6H_2_O, DPPH, Trolox, gallic acid, and catechin were purchased from Sigma-Aldrich Pty Ltd. (Castle Hill, Sydney, Australia). HCl and KCl were purchased from ChemSupply (Port Adelaide, SA, Australia). K_2_S_2_O_5_ was purchased from Histolabs (Riverstone, NSW, Australia). Colourless, flat-bottom microplates were used in the spectrophotometric assays. All dilutions were made using deionised water unless otherwise specified.

The Illawarra plums (*Podocarpus elatus*) were harvested from Newcastle, NSW, Australia (Latitude: S32°53′40.67″ Longitude: E151°43′17.87″). The plant was authenticated by a botanist at the University of Newcastle, and a sample was deposited in the University of Newcastle herbarium (voucher number 10764). External seeds were separated from the fruit by twisting. Whole fruits were stored at −18 °C prior to processing.

Frozen fruits were halved using a chef’s knife, de-seeded, and then dried cut-side up in a single layer on clean, pre-dried foil trays, as per McCullum et al. [19]. Vacuum drying was undertaken at 90 °C (VD) and 450 Torr vacuum pressure for 2.75 h (Thermoline Vacuum drier, Marrickville, NSW, Australia) McCullum et al. [19]. Freeze drying (FD) conditions were −45 °C and 135 mTorr for 48 h (Benchtop Pro Freeze Drier, Scitek, Lane Cove, NSW, Australia). After drying, the samples were ground to a powder using a spice grinder (Breville BCG200BSS, Villawood, NSW, Australia) and then stored at −18 °C under airtight conditions.

VD and FD Illawarra plum extracts (VD ext and FD ext) were prepared using ultrasonic-assisted extraction for 60 min at 40 °C, 150 W, using 50% aqueous ethanol at a sample–solvent ratio of 1:100 g/mL (Soniclean, 220 V, 50 Hz, 250 W, Thebarton, Australia). Following extraction, the samples were cooled in an ice bath and then centrifuged at 3100× *g* for 5 min. The supernatant was collected, and the solvent was removed by rotary evaporation (40 °C water bath, 60 mBar). The extracts were then freeze dried (−45.2 °C 236 mTorr), and the resulting powder extract was stored at −18 °C.

To prepare the yoghurts, a commercially available EasiYo Unsweetened Greek-style set yoghurt base was used. It contained 99% milk solids, emulsifier (soy lecithin), and live yoghurt cultures (80 billion cfu/100 g) of *L. bulgaricus*, *S. thermophilus*, *L. acidophilus*, and *L. rhamnosus*. The commercial yoghurt base (powder) was mixed with powdered extract (VD ext and FD ext) or dried ground fruit (VD and FD) and hydrated with deionised water to a final concentration of 17% yoghurt base and 0.5% Illawarra plum. The mixture was incubated at 35 °C for 18 h and then transferred to chilled storage.

Assessment of quality parameters was conducted during chilled storage (1–4 °C) on days 1, 3, 5, 7, 10, 12, 14, and 28.

Syneresis (%) as free whey was determined and calculated according to the method described by [20,21] with the following modifications. Samples were centrifuged at 4 °C and 3100× *g* for 20 min (Centrifuge 5804R, Eppendorf SE, Hamburg, Germany). Titratable acidity was determined by titrating diluted yoghurt with 0.1 M NaOH using a phenolphthalein indicator to a colourimetric endpoint [22,23]. Lactic acid equivalent weight of 90 meQ was used in the calculation, and the data was presented as lactic acid mg/100 g [22,23]. Total solids content was recorded as Brix % using a digital hand-held refractometer (Pal-1, Atago, Tokyo, Japan). Colour was measured in L*, a* and b* units by a colourimeter (Chromameter CR-20 Konika Minolta, Tokyo, Japan). pH was recorded by electrode reading (Smart CHEM-Lab benchtop pH meter, TPS, Brendale, QLD, Australia).

Qualitative analysis of phenolics, flavonoids, monomeric anthocyanins, antioxidant scavenging activity by DPPH, and proanthocyanins (tannins) during storage was determined by spectrophotometric methods including the Folin Ciocalteu assay, as described by McCullum et al. [19]. Briefly, the colourimetric response was defined as strong +++, moderate ++, or minor +. Where no response was observed, these were recorded as ‘−’ [24,25]. Positive controls of gallic acid, catechin, and trolox were used for phenolic, flavonoid and proanthocyanin, and DPPH assessment, respectively.

**Figure 1:**
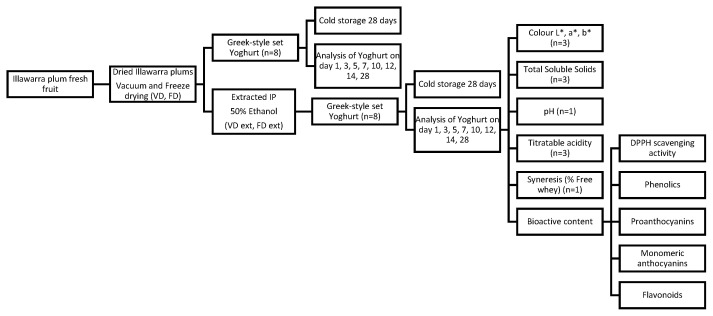
Overview of study design to assess the quality of yoghurt made with dried or dried and extracted Illawarra plum.

Yoghurts were prepared as replicates (*n* = 8) of 50 mL volume to allow for an undisturbed sample to be assessed at each time point during storage as per the study design in Figure 1. The goal of this was to prevent contamination of the sample during storage and prevent any influence from fluctuating temperatures and environmental exposure during the storage period. On day 28, both a replicate sample and the same sample from day 1 were assessed to allow for a direct comparison between a single sample on day 1 and day 28 of storage.

To allow for the calculation of mean values, a minimum of three separate measurements were recorded for colour, total solids, qualitative biochemical screening and titratable acidity for each replicate. pH and syneresis were measured only once for each replicate. Tabulated data was calculated as the means ± standard errors of the replicate measurements. Graphical data was calculated as the mean value unless otherwise written and presented with a spline smoother (lambda given in figure caption). Kernel smoothing is widely used in nonparametric estimation to reduce the impact of natural variation from time-series errors and repeated measurements [26].

One-way ANOVA using Tukey’s post-hoc comparison of means was performed to assess statistical differences (*p* < 0.05) between samples or time points. Statistical analyses were completed using JMP Pro 17.2.0 statistical software (JMP 2022-2023, JMP Statistical Discovery LLC, New York, NY, USA).

## 3. Results

### 3.1. Colour of Yoghurts Fortified with Illawarra Plum during 28-Day Chilled Storage

**Figure 2:**
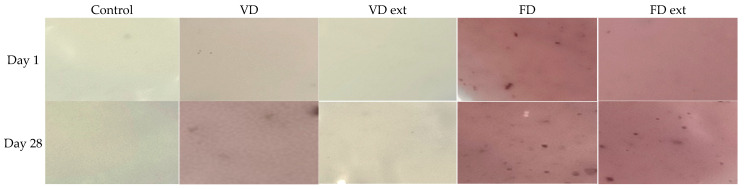
Colour of yoghurts (Control and fortified with IP and IP extract) on day 1 and day 28 of storage. FD: Freeze dried; VD: Vacuum dried.

The colour of the yoghurts ranged from creamy white to dark pink (Figure 2). On day 1 of storage, all yoghurt fortified with the Illawarra plum had a significantly different colour to the control (*p* < 0.05).

The control yoghurt was the li ghtest and whitest, as indicated by the highest mean L* of 91.49 +/− 0.18, b* of 14.06 +/− 0.24 and a* −1.32 +/− 0.04 (Table 1). The yoghurts fortified with FD and FD extracts had a significant difference in colour as compared to the control but were very similar to each other in terms of redness and yellowness (*p* < 0.05) (Table 1). The yoghurts fortified with VD extract had a* values which changed from negative values, or very slight greenness, in the first 10 days of storage, to positive values, or no greenness and slight redness, from day 10 to 28. There was little difference in the colour density of samples between day 1 and day 14, except for VD at day 1.

### 3.2. Total Soluble Solids of the Yoghurts Fortified with Illawarra Plum during Storage

**Figure 3:**
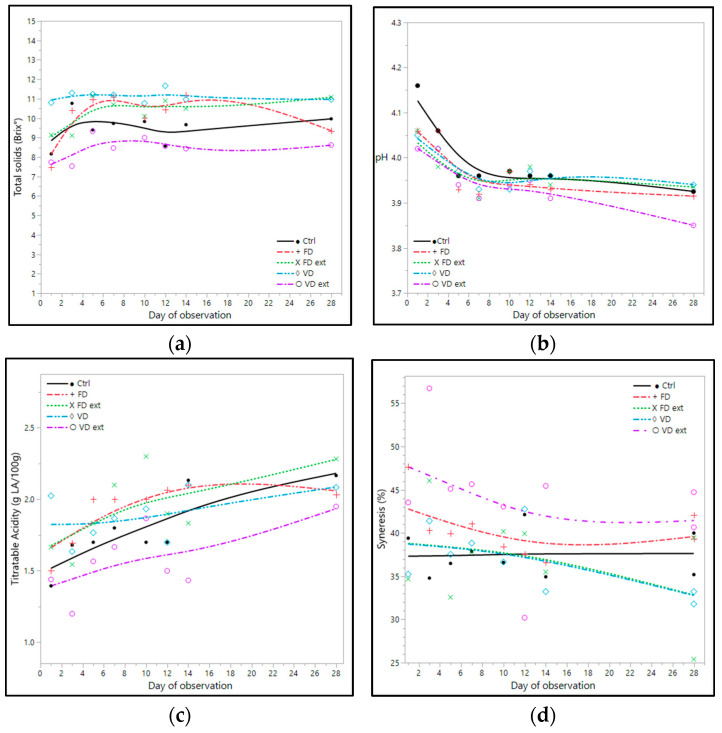
Impact of IP fruit and fruit extract on (**a**) total soluble solids; (**b**) pH; (**c**) titratable acidity; and (**d**) syneresis (% free whey) of yoghurts. IP yoghurts were made using different processing methods: VD: vacuum-dried Illawarra, VD ext: vacuum-dried and extracted, FD: freeze dried, and FD ext: freeze-dried and extracted Illawarra plum fruits. Ctrl: control yoghurt. LA: lactic acid. Where available, data is the mean ± std error (*n* = 3). Spline smoothing was applied with lambda values of (**a**) 0.05, (**b**) 0.05, (**c**) 1.75, and (**d**) 1.75.

In general, the yoghurts fortified with the Illawarra plum had higher levels of solids than the control (Figure 3a). The yoghurt fortified with VD fruit had the highest mean solids (11.10 ± 0.12 °Brix), followed by the yoghurt fortified with FD extract (10.42 ± 0.19 °Brix) Table 2. The control and the yoghurt fortified with VD extract had the lowest solids. Overall, soluble solids of all yoghurts increased between days 1 and 28 of storage (Figure 3a). Soluble solids of the control increased approximately 1.8 °Brix over the 28 days of storage, but the yoghurts fortified with the Illawarra plum had less increase in levels of solids in comparison with the control in the same timeframe (Figure 3a).

### 3.3. pH and Titratable Acidity of the Yoghurts Fortified with Illawarra Plum during Storage

All yoghurts had similar acidity with pH ranging from 3.93 to 3.98 and mean titratable acidity between 1.62 ± 0.05 and 1.97 ± 0.07 g lactic acid/100 g, as shown in Table 2. The pH of all yoghurts fortified with the Illawarra plum on day 1 was lower by more than 0.10 than the control yoghurt. The pH of all the yoghurts decreased in a range of 0.11–0.24 between days 1 and 28 (Figure 3b). Notably, the greatest reduction in pH occurred in the first 5 days of storage for all samples. The titratable acidity reflects the changes in pH and increased with increasing storage time for all samples (Figure 3c). On day 28, there was no significant difference (*p* < 0.05) in the titratable acidity between any of the IP yoghurts or the control.

### 3.4. Syneresis of Yoghurts during Storage

The yoghurt made with VD extract had the highest mean syneresis at 43.90 ± 2.27% free whey, and the lowest was in the yoghurt made with VD at 36.74 ± 1.26 (Table 2). Syneresis of all yoghurts tended to decrease during storage, in which all the yoghurts fortified with the Illawarra plum had lower syneresis on day 28 in comparison with day 1 (Figure 3d). The degree of change between days 1 and 28 was between 0.84 and 6.96%. The yoghurts fortified with VD, FD, and FD extract had similar mean syneresis to the control yoghurt (*p* > 0.05).

### 3.5. Qualitative Phytochemical Screening of Yoghurts

The phytochemicals and antioxidant activity were assessed at regular intervals during storage, and the results are shown in Table 3. All yoghurts, including the control, contained phenolics. During the 28 days of storage, the yoghurts fortified with FD and FD extract had the strongest response of phenolics and antioxidants of all the yoghurts, and the VD ext yoghurt had the least phenolics. 

The results showed that yoghurts fortified with FD and FD extract had the greatest level of anthocyanins, whereas yoghurts fortified with VD and VD extract had the least anthocyanins.

The yoghurt fortified with FD had moderate levels of flavonoids, anthocyanins, and proanthocyanins. The yoghurt fortified with FD extract had a low to moderate response for flavonoids, anthocyanins, and proanthocyanins throughout the storage period, indicating a reduced amount compared with the yoghurt fortified with FD.

Scavenging activity of the yoghurts was observed in FD, FD ext, and VD up to and including day 28. In comparison, the VD ext and control yoghurts did not show any response to the DPPH radical scavenging assay on days 1, 7, 14, or 28. There was little to no change in anthocyanin response for VD, FD, and FD ext yoghurts throughout the storage period.

## 4. Discussion

The physicochemical parameters of yoghurt are objective measures of quality that can directly impact the sensory experience. Sensory quality can be preliminarily gauged by physicochemical attributes such as colour, total solids, acidity, and syneresis.

The colour of yoghurt is an important aspect and will affect how acceptable it is to consumers [27]. In this study, the FD, FD extracted, and to a lesser extent, VD Illawarra plum contributed an attractive pink colour to Greek-style set yoghurt. As expected, the levels of anthocyanin reflect the colour intensity of the yoghurts, with the samples that had higher anthocyanins having a richer pink colour. None of the IP yoghurts had negative values for b*, indicating no blueness and that the IP yoghurts are indeed pink and not purple. In terms of colour intensity for redness, from most to least, the yoghurts can be ranked FD ext >FD > VD > VD ext > Ctrl. From darkest to lightest, the yoghurts can be ranked FD/FD ext > VD > VD ext > Ctrl. This is consistent with other studies of yoghurts made with extracts from different anthocyanin-rich fruits, such as red grape varieties and honeysuckle berry, which were light red or purple-coloured yoghurts [7,27,28,29]. However, in these studies, the level of anthocyanins decreased over 7 or 14 days of storage [7,27,28,29]. In this study, the yoghurts fortified with FD and FD-extracted Illawarra plum showed no significant difference between colour values for L* and a* between day 1 and day 28 of storage. This indicates there was reasonable stability of the colours in this formulation. Degradation of anthocyanins occurs due to the presence of destructive enzymes, such as glycosidase, increased oxygen incorporation, or changes in pH, heat and light [27,28,29]. There was little to no change in anthocyanins in the VD, FD, and FD ext IP yoghurts in the current study. This can be explained by the type of yoghurt, process, and storage conditions used in this study. A set-yoghurt was incubated in the dark and stored in tightly sealed sample containers in a dark fridge. For each day of analysis, a new replicate was used which minimised oxygen exposure. If a stirred type of yoghurt was chosen, it would be expected there would be more oxygen incorporation. Thus, there was better protection of the anthocyanins from deterioration by oxygen in this study.

Soluble solid content can help to obtain consistency and consumer acceptance of yoghurt. Higher levels of total solids in yoghurts increase oral viscosity or perceived thickness, increase gel firmness, reduce syneresis, and improve texture acceptability for consumers [30,31,32]. The current results revealed that VD, FD, and FD-extracted Illawarra plum increased the soluble solid content of the yoghurt compared to the control and showed an increase in solids over time during storage. The higher solids compared to the control is attributed to the inclusion of the plum. The observed increase in soluble solids in IP yoghurts between days 1 and 28 can be explained by a loss in moisture content during storage [33]. This has been seen in other yoghurt fortification studies [34,35]. From the changes in solids, it can be presumed that the VD, FD, and FD-extracted yoghurt improved the yoghurt’s consistency. There is a limitation to the study in that the tests are objective and that consumer preference, acceptability and, therefore, acceptance of a fortified yoghurt will ultimately rely on subjective sensory assessments. The impact of the Illawarra plum addition to consumer acceptance and preference of the yoghurts should be pursued in future studies.

Acidity is an important quality parameter and contributes to the perceived sourness, stability, and food safety of yoghurts. In the yoghurts, there was a drop in pH during the first 5 days of storage and this can be considered the result of residual lactose fermentation or ‘post-acidification’. Acid-tolerant *Bacillus* spp. can continue to produce lactic acid during chilled storage [3,17,21,25,26]. In set-type yoghurt, as used in this study, the greatest changes in pH and acidity occur in the first 7 days of storage [25,26]. Iranian yoghurts observed a reduction in pH only during the first week of storage [27]. In that study by Szoltysik et al. [27], the pH of a yoghurt made with a berry extract continued to decrease for the 2 weeks of storage. Similarly, the pH of yoghurt made with fennel essential oil continued to decrease throughout its 28-day storage [21]. The degree of post-acidification might cause negative effects on texture or stability, increasing firmness and whey syneresis [5]. The IP yoghurts did not continue to drop in the same increments of pH after day 5, indicating post-acidification did not continue, and there was no significant difference (*p* < 0.05) between all the yoghurts at day 28. These results indicate that the acidity and the sourness of the yoghurt remain unchanged by the fortification with IP.

A study on the fortification of yoghurt with chamomile or fennel observed a similar finding in that these additives did not significantly affect the pH. This is important for controlling safety and preventing the growth of *Listeria monocytogenes*, *Salmonella* spp., and *E. coli* Caleja et al. [36]. The optimal pH for yoghurt and acceptability of sourness perception is 4–4.4 [5]. The IP yoghurts, which have a mean pH of just under 4.0, showed a slight increase in acidity during the 28 days of storage. In another study, the increase in acidity during storage in a yoghurt fortified with honey decreased scores for sourness as rated by a sensory panel [33]. From this, it can be inferred that there could be an overall unfavourable sourness in the IP yoghurts. However, this study used a Greek-style starting culture and an 18 h incubation time; thus, a reasonable level of sourness is expected. A study of sensory drivers of liking for Greek yoghurt indicated that consumers prefer a moderate sour taste in this style of yoghurt [37]. It could be expected that a reduction in incubation time could slightly reduce the acidity of the final product [30]. The time to reach optimal pH to understand how the addition of IP impacts the yoghurt throughout the fermentation process could be a future avenue for research. Further, this study only investigated one combination of starter cultures, and the type, amount of inoculum, and composition of starting ingredients could also influence the final acidity of the finished yoghurt, warranting further investigation.

The syneresis of yoghurt can be influenced by pH, nutritional or nutraceutical composition (pectin, phenolics), and total solids. A reduction in pH causes contraction of the casein micelles within the yoghurt emulsion and results in the expulsion of liquid whey to the top of the yoghurt [29]. In the Illawarra plum yoghurts, there was little change in pH at day 28 compared to the control. Further, there was no significant difference in mean syneresis of the VD, FD, and FD ext yoghurts compared to the control yoghurt. The IP yoghurt made with VD ext, which observed the highest syneresis, had the lowest pH of all the yoghurts. In a study by Gurkan et al. [38], yoghurt made with purple basil powder showed increasing levels of syneresis during 21 days of storage, combined with a reduction in pH. Conversely, an Iranian yoghurt increased pH during storage, and the level of syneresis reduced [31]. There was a trend towards decreased syneresis on day 28 compared to day 1 for all yoghurts; however, based on the sampling method, there was not enough data for statistical comparison between each storage day to understand the significance of this trend. There was no negative effect on the synthesis of yoghurts made with VD, FD, or FD ext Illawarra plum.

The syneresis of yoghurt is also thought to be reduced by phenolics stabilising milk proteins via electrostatic interactions or hydrogen bonds [39]. Each yoghurt contained phenolics, including the control, and the VD ext yoghurt with the least phenolics had significantly higher mean syneresis than the control yoghurt. The IP yoghurts in this study were made with 0.5% fortification, and it is expected that the phytochemical activity would increase in proportion to the fortificant [40]. Pectin may also contribute to syneresis and the body of the yoghurt, as suggested by Dlamini et al. [41], who added an indigenous African fruit to yoghurt and observed it prevented the potential for syneresis compared to plain or strawberry yoghurt. Australian native plums contain pectin as a main component in the cell wall polysaccharides [42]. The effect of increased concentrations of IP might also increase pectic substances and improve syneresis outcomes for the yoghurt and could be an area for further investigation.

The presence of phytochemicals, such as phenolics and anthocyanins, contributes to the physicochemical quality already described for colour or syneresis but will also improve the functional quality of yoghurt. Functional quality refers to the potential for improving the health benefits of the food beyond providing basic nutrition. Phenolics may provide functional benefits, including improving outcomes for inflammation, clotting, cardiovascular disease or cancer, and incorporation into yoghurt can add to the benefits of probiotics for chronic diseases, including high blood pressure, intestinal disorders, and high cholesterol [9,43]. However, the potential for this activity is not yet proven, and for it to be of any benefit, the stability and presence of the phytochemicals in processed food products need to be understood.

The addition of plant extracts is known to contribute antioxidant activity to products, and anthocyanins have a positive correlation with antioxidant activity [27]. The results revealed that most of the yoghurts fortified with the Illawarra plum exhibited antioxidant activities, except for the one fortified with VD extract. In contrast, no antioxidant activity was observed for the control. The level of phenolics in FD and FD ext IP yoghurts remained high at 28 days, which is similar to a hibiscus-infused yoghurt, which showed no statistical difference in phenolic levels after 14 days of storage [29]. The antioxidant properties in the yoghurts fortified with VD, FD, and FD extract of IP decreased slightly towards the end of the storage period. This is consistent with the reduction in antioxidant activity in yoghurt made with red dragon fruit by Gengatharan et al. [39] and honeysuckle berry by Szoltysik et al. [27]. Despite the reduction in antioxidants, there is still a moderate presence of active radical scavenging activity in the FD and FD ext yoghurt on day 28. This offers promise to the functional potential of IP as a fortificant for the functional benefit of yoghurt. Further studies are suggested to study the levels of antioxidants, such as flavonoids, anthocyanins and their correlations with antioxidant properties.

From this study, the quality of yoghurt fortified with VD, FD, and FD-extracted IP was assessed. The colour provided by anthocyanins in the FD and FD ext yoghurts and increased levels of phytochemicals in these samples made the FD and FD ext IP superior compared to the VD yoghurt for the purpose of improving the quality. There were slight differences between FD and FD ext. However, the cost benefit of an additional step of extraction and solvent removal could not be justified. The FD ext was not as efficient or cost-effective, nor did it significantly improve the outcomes in the finished yoghurt during its 28-day storage. Therefore, FD IP is recommended over its extract for fortifying yoghurt.

## 5. Conclusions

Illawarra plum freeze-dried powder and its extract can be fortified in Greek-style set yoghurt to improve its colour, total solids, and phytochemicals during chilled storage for up to a month. This study recommended fortifying the Greek-style set yoghurt with Illawarra plum powder prepared from freeze-drying fresh fruits. The anthocyanins in the Illawarra plum powder contribute a stable, distinct pink colour to the yoghurt. It also could improve solid content and, thus, the consistency of yoghurt. In addition, fortification of Greek-style set yoghurt with this Illawarra plum powder did not affect its acidity and syneresis during chilled storage. While it can increase the level of phenolics, anthocyanins, and antioxidant properties of the yoghurt, the functional benefit has yet to be investigated. The results of the study revealed a great opportunity for dried IP powder as an added functional ingredient and colourant. To elucidate its potential use, future studies are needed to evaluate subjective sensory quality and consumer responses and prove a functional benefit of Greek-style set yoghurt fortified with FD IP powder. 

## Figures and Tables

**Table 1:** Impact of IP fruit and fruit extract on the colour of yoghurts during 28 days of storage.

Day of Storage			Control	VD	VD ext	FD	FD ext
			L* (Lightness)				
*n* = 3	1		90.9 ± 0.26 ^1ab^	86.0 ± 0.15 ^3a^	88.9 ± 0.12 ^2bc^	64.4 ± 0.21 ^5a^	69.1 ± 0.12 ^4cd^
	3		89.9 ± 0.53 ^1b^	76.4 ± 0.48 ^3b^	87.7 ± 0.32 ^2c^	62.7 ± 0.10 ^5a^	71.1 ± 0.06 ^4b^
	5		92.5 ± 0.17 ^1a^	79.1 ± 0.38 ^3b^	89.8 ± 0.15 ^2ab^	61.2 ± 0.06 ^5a^	71.0 ± 0.12 ^4b^
	7		91.2 ± 0.44 ^1ab^	74.9 ± 0.93 ^2b^	89.2 ± 0.32 ^1abc^	60.7 ± 0.12 ^4a^	71.8 ± 0.20 ^3ab^
	10		92.3 ± 0.03 ^1a^	75.7 ± 0.94 ^2b^	90.9 ± 0.24 ^1a^	61.4 ± 0.24 ^4a^	72.6 ± 0.15 ^3a^
	12		92.0 ± 0.18 ^1a^	77.4 ± 1.54 ^2b^	88.9 ± 0.12 ^1abc^	62.6 ± 1.06 ^4a^	69.5 ± 0.32 ^3c^
	14		92.2 ± 0.27 ^1a^	75.2 ± 1.28 ^3b^	88.7 ± 0.30 ^2bc^	61.4 ± 0.12 ^5a^	71.5 ± 0.12 ^4ab^
	28		91.2 ± 0.29 ^1ab^	75.9 ± 1.03 ^2b^	89.0 ± 0.50 ^1bc^	61.7 ± 1.04 ^4a^	68.4 ± 0.25 ^3d^
Mean (*n* = 8)			91.49 ± 0.18 ^a^	77.39 ± 0.31 ^c^	89.12 ± 0.29 ^b^	61.98 ± 0.71 ^e^	61.98 ± 0.20 ^d^
			a* (red+/green−)				
*n* = 3		1	−1.3 ± 0.12 ^4ab^	3.8 ± 0.27 ^2c^	−0.2 ± 0.12 ^3bc^	16.4 ± 0.10 ^1a^	16.5 ± 0.15 ^1ab^
		3	−1.5 ± 0.19 ^4ab^	9.6 ± 0.12 ^3ab^	−0.8 ± 0.13 ^4c^	14.0 ± 0.38 ^2b^	15.9 ± 0.09 ^1ab^
		5	−1.1 ± 0.03 ^3ab^	9.0 ± 0.64 ^2ab^	−0.8 ± 0.09 ^3c^	16.0 ± 0.38 ^1ab^	16.9 ± 0.07 ^1ab^
		7	−1.4 ± 0.07 ^5ab^	10.6 ± 0.15 ^6a^	−0.5 ± 0.12 ^7c^	16.8 ± 0.24 ^4a^	16.0 ± 0.12 ^2ab^
		10	−1.3 ± 0.03 ^3ab^	8.9 ± 0.48 ^2ab^	−0.1 ± 0.03 ^3bc^	16.1 ± 0.36 ^1ab^	16.1 ± 0.25 ^1ab^
		12	−1.3 ± 0.06 ^4ab^	8.5 ± 0.22 ^2b^	1.6 ± 0.03 ^3a^	16.7 ± 0.62 ^1a^	17.6 ± 0.38 ^1a^
		14	−1.6 ± 0.07 ^5b^	8.9 ± 0.26 ^3ab^	1.2 ± 0.15 ^4a^	16.7 ± 0.31^1a^	15.3 ± 0.10 ^2b^
		28	−1.2 ± 0.06 ^5a^	10.0 ± 0.38 ^3ab^	0.8 ± 0.34 ^4ab^	15.2 ± 0.48 ^2ab^	17.0 ± 0.47 ^1a^
Mean (*n* = 8)			−1.32 ± 0.04 ^d^	8.79 ± 0.39 ^b^	0.23 ± 0.19 ^c^	15.9 ± 0.22 ^a^	16.48 ± 0.17 ^a^
			b* (yellow+/blue−)				
*n* = 3		1	15.2 ± 0.50 ^1a^	9.0 ± 0.09 ^3a^	12.5 ± 0.34 ^2ab^	4.0 ± 0.22 ^4a^	0.8 ± 0.00 ^5d^
		3	12.9 ± 1.25 ^1a^	3.3 ± 0.15 ^2b^	11.0 ± 1.06 ^1b^	1.2 ± 0.28 ^2c^	1.7 ± 0.03 ^2bcd^
		5	15.6 ± 0.10 ^1a^	4.5 ± 0.12 ^3b^	11.7 ± 0.54 ^2b^	1.2 ± 0.17 ^4c^	2.3 ± 0.07 ^4abc^
		7	13.2 ± 0.49 ^1a^	3.1 ± 0.31 ^2b^	11.3 ± 0.68 ^1b^	1.2 ± 0.15 ^2c^	2.8 ± 0.15 ^2ab^
		10	14.3 ± 0.23 ^1a^	2.9 ± 0.47 ^2b^	14.9 ± 0.15 ^1a^	1.4 ± 0.15 ^3bc^	3.1 ± 0.15 ^2a^
		12	13.8 ± 0.49 ^1a^	3.6 ± 0.42 ^3b^	11.8 ± 0.03 ^2b^	2.4 ± 0.58 ^3bc^	2.3 ± 0.13 ^3a^
		14	13.4 ± 0.65 ^1a^	2.9 ± 0.12 ^2b^	12.8 ± 0.42 ^1ab^	1.2 ± 0.15 ^3c^	2.8 ± 0.03 ^2,3abc^
		28	14.1 ± 0.38 ^1a^	3.6 ± 0.35 ^3b^	11.8 ± 0.26 ^2b^	2.4 ± 0.14 ^3,4b^	1.8 ± 0.31 ^4c^
Mean (*n* = 8)			14.06 ± 0.24 ^a^	4.07 ± 0.37 ^c^	12.17 ± 0.26 ^b^	1.94 ± 0.19 ^d^	2.17 ± 0.15 ^d^

Data is presented as the mean +/− std error of three measurements of a yoghurt sample. Within rows of a colour component, values with different superscript numbers are significantly different (*p* < 0.05). Within columns of a colour component, values with a different superscript letter are significantly different (*p* < 0.05). Within a colour component, values with different superscript letters are statistically different (*p* < 0.05). Yoghurts are made with FD (freeze dried fruit powder); FD ext (freeze dried, extracted fruit); VD (vacuum-dried fruit powder); VD ext (vacuum-dried, extracted fruit).

**Table 2:** Impact of IP fruit and fruit extract on solids, pH, titratable acidity, and syneresis of yoghurts on days 1, 7, 14, and 28 of storage.

Day of Storage		Control	Vacuum Dried	VD Extracted	Freeze Dried	FD Extracted
Total Soluble Solids (°Brix) *n* = 3	1	8.17 ± 0.07 ^c^	10.80 ± 0.00 ^a^	7.73 ± 0.09 ^cd^	7.47 ± 0.09 ^d^	9.13 ± 0.18 ^b^
	7	9.73 ± 0.39 ^b^	11.20 ± 0.30 ^a^	8.47 ± 0.07 ^c^	11.10 ± 0.15 ^a^	10.70 ± 0.26 ^ab^
	14	9.67 ± 0.09 ^bc^	10.97 ± 0.41 ^ab^	8.43 ± 0.03 ^c^	11.20 ± 0.25 ^a^	10.50 ± 0.50 ^ab^
	28	9.97 ± 0.30 ^b^	10.97 ± 0.13 ^a^	8.62 ± 0.07 ^c^	9.35 ± 0.17 ^bc^	11.08 ± 0.27 ^a^
Mean		9.56 ± 0.16 ^c^	11.10 ± 0.12 ^a^	8.48 ± 0.11 ^d^	10.03 ± 0.23 ^bc^	10.42 ± 0.19 ^b^
Titratable Acidity (g LA/100 g) *n* = 3	1	1.40 ± 0.00 ^c^	2.03 ± 0.03 ^a^	1.44 ± 0.00 ^c^	1.50 ± 0.05 ^c^	1.67 ± 0.03 ^b^
	7	1.80 ± 0.15 ^ab^	1.87 ± 0.03 ^ab^	1.67 ± 0.03 ^b^	2.00 ± 0.00 ^ab^	2.10 ± 0.06 ^a^
	14	2.13 ± 0.34 ^a^	2.10 ± 0.10 ^a^	1.43 ± 0.07 ^a^	2.10 ± 0.06 ^a^	1.83 ± 0.09 ^a^
	28	2.17 ± 0.15 ^a^	2.08 ± 0.06 ^a^	1.95 ± 0.03 ^a^	2.03 ± 0.08 ^a^	2.28 ± 0.18 ^a^
Mean		1.83 ± 0.07 ^a^	1.91 ± 0.04 ^a^	1.62 ± 0.05 ^b^	1.94 ± 0.04 ^a^	1.97 ± 0.07 ^a^
pH *n* = 1	1	4.16	4.05	4.02	4.06	4.06
	7	3.96	3.93	3.91	3.92	3.91
	14	3.96	3.96	3.91	3.94	3.98
	28	3.93	3.94	3.85	3.92	3.94
Mean		3.98 ± 0.02 ^a^	3.96 ± 0.02 ^a^	3.93 ± 0.02 ^a^	3.96 ± 0.02 ^a^	3.96 ± 0.01 ^a^
Syneresis (% free whey) *n* = 1	1	39.40	35.24	43.54	47.67	34.67
	7	37.86	38.84	45.65	41.06	37.79
	14	34.94	33.23	45.46	36.62	35.49
	28	37.60	32.51	42.69	40.71	32.43
Mean		37.49 ± 0.85 _b_	36.74 ± 1.26 ^b^	43.90 ± 2.27 ^a^	40.34 ± 1.08 ^ab^	36.84 ± 1.93 ^b^

Within a row, values not sharing a superscript letter are significantly different (*p* < 0.05). LA = Lactic acid.

**Table 3:** Impact of IP fruit and fruit extracts on phytochemicals of yoghurt during 28 days storage.

Type of Yoghurt	Day of Storage	Phenolics	Flavonoids	Proantho	DPPH	Antho
Vacuum-Dried Illawarra plum	1	++	+	+	++	+
	3	++	−	+	−	+
	5	++	−	+	++	+
	7	++	+	+	+	+
	10	++	+	+	+	+
	12	++	+	+	+	+
	14	+	−	+	+	−
	28	++	−	+	+	+
Vacuum-Dried Extract of Illawarra plum	1	++	−	−	−	−
	3	+	−	−	−	−
	5	++	−	−	+	−
	7	+	−	−	−	−
	10	++	−	−	−	−
	12	+	−	−	−	−
	14	+	−	−	−	−
	28	++	−	−	−	−
Freeze-Dried Illawarra plum	1	+++	++	++	++	+
	3	+++	++	++	+	++
	5	+++	++	++	+++	++
	7	+++	++	++	+	++
	10	+++	++	++	+++	++
	12	+++	++	++	++	++
	14	+++	++	++	++	++
	28	+++	++	+	++	++
Freeze-Dried Extract of Illawarra plum	1	+++	++	++	++	++
	3	+++	+	++	−	++
	5	+++	++	+	+++	+
	7	+++	+	+	+	+
	10	+++	+	+	++	+
	12	+++	+	++	++	++
	14	++	++	+	+	+
	28	+++	++	++	++	++
Control	1	++	−	−	−	−
	3	+++	−	−	−	−
	5	++	−	−	−	−
	7	+	−	−	−	−
	10	++	−	−	−	−
	12	+	−	−	−	−
	14	+	−	−	−	−
	28	++	−	−	−	−

Proantho: Proanthocyanins. Antho: monomeric anthocyanins. “+++” indicates a strong response; “++” indicates a moderate response; “+” indicates a minor response; “−” Indicates no response in the assay.

## Data Availability

The original contributions presented in the study are included in the article, further inquiries can be directed to the corresponding author.
